# Therapeutic Time Window for Edaravone Treatment of Traumatic Brain Injury in Mice

**DOI:** 10.1155/2013/379206

**Published:** 2013-04-10

**Authors:** Kazuyuki Miyamoto, Hirokazu Ohtaki, Kenji Dohi, Tomomi Tsumuraya, Dandan Song, Keisuke Kiriyama, Kazue Satoh, Ai Shimizu, Tohru Aruga, Seiji Shioda

**Affiliations:** ^1^Department of Anatomy, Showa University School of Medicine, 1-5-8 Hatanodai, Shinagawa-ku, Tokyo 142-8555, Japan; ^2^Department of Emergency and Critical Care Medicine, Showa University School of Medicine, 1-5-8 Hatanodai, Shinagawa-ku, Tokyo 142-8555, Japan

## Abstract

Traumatic brain injury (TBI) is a major cause of death and disability in young people. No effective therapy is available to ameliorate its damaging effects. Our aim was to investigate the optimal therapeutic time window of edaravone, a free radical scavenger which is currently used in Japan. We also determined the temporal profile of reactive oxygen species (ROS) production, oxidative stress, and neuronal death. Male C57Bl/6 mice were subjected to a controlled cortical impact (CCI). Edaravone (3.0 mg/kg), or vehicle, was administered intravenously at 0, 3, or 6 hours following CCI. The production of superoxide radicals (O_2_^∙−^) as a marker of ROS, of nitrotyrosine (NT) as an indicator of oxidative stress, and neuronal death were measured for 24 hours following CCI. Superoxide radical production was clearly evident 3 hours after CCI, with oxidative stress and neuronal cell death becoming apparent after 6 hours. Edaravone administration after CCI resulted in a significant reduction in the injury volume and oxidative stress, particularly at the 3-hour time point. Moreover, the greatest decrease in O_2_^∙−^ levels was observed when edaravone was administered 3 hours following CCI. These findings suggest that edaravone could prove clinically useful to ameliorate the devastating effects of TBI.

## 1. Introduction

In spite of the fact that traumatic brain injury (TBI) is a major cause of death and disability, particularly in young people, and given the huge socioeconomic costs of caring for affected persons, there is still no adequate treatment available to ameliorate its damaging effects [1, 2]. The overall incidence of TBI in the United States is estimated to be 540 cases per 100,000 persons and the prevalence of long-term disability is estimated to be between 3.2 and 5.3 million. In 2000, the economic impact of TBI in the United States was estimated to be $9.2 billion in lifetime medical costs and $51.2 billion in lost productivity. Falls and motor vehicle accidents are the leading causes of TBI, with most cases transferred immediately to an emergency department [3]. Given that moderate and severe TBIs are associated with neurologic and functional impairments [4], further intensive care following initial treatment and diagnostic assessment are also usually required.

Edaravone (3-methyl-1-phenyl-2-pyrazolin-5-one) is a derivative of antipyrin and was approved as free radical scavenger for the treatment of acute cerebral infarction in Japan [5]. Edaravone was first reported to strongly scavenge hydroxyl radicals (OH^−^) produced by the Fenton reaction *in vitro* and to decrease lipid and L-tyrosine oxidation [6]. The effects of edaravone have been studied in relation to brain ischemia in animals and humans, and decreased brain edema, infarction, endothelial damage, and oxidative damage have been reported [6–11]. Edaravone has also been used in other neural injury models such as spinal cord injury [12], TBI [8, 13, 14], and brain hemorrhage [15] and was found to reduce lesion size and oxidative stress levels. We have previously reported that intravenous edaravone (3.0 mg/kg) treatment immediately after cortical impact suppressed traumatic neural damage in rodents [13] and decreased hydroperoxide (ROO^*∙*^) and alkoxyl (RO^*∙*^) radical formation in both rodents and patients with TBI [13, 16]. Edaravone treatment at 2 hours and again at 12 hours also decreased neuronal loss in a dose-dependent fashion (0.75, 1.5, or 3 mg/kg) in the CA3 layer of the hippocampus after TBI [8], while at 3 mg/kg i.v it suppressed apoptotic neuronal cell death and oxidative damage after TBI [14]. In spite of these positive outcomes, the therapeutic time window of edaravone on TBI has not been examined in detail.

It has been suggested that reactive oxygen species (ROS) generation is activated in the lesion area after TBI, leading to the initial production of superoxide (O_2_^∙−^) and nitric oxide (^*∙*^NO) radicals. These ROS then react and metabolize to form stronger oxidants in the form of peroxynitrite (ONOO^−^), hydroxyl (^*∙*^OH), carbonate (CO_3_^∙−^), and nitrogen dioxide (^*∙*^NO_2_) radicals [17, 18], which in turn react with proteins, lipids, sugars, and nucleotides and impair the normal physiological function of cells. Although it is considered that O_2_^∙−^ does not have strong oxidative potential and that edaravone does not scavenge O_2_^∙−^ *in vitro* [6], we previously reported that mice deficient in Gp91^phox^ (NOX2), a subunit of NADPH oxidase and a generator of O_2_^∙−^, exhibited reduced lesion size and oxidative stress following TBI [19]. Moreover, knockout mice lacking interleukin-1, a proinflammatory cytokine, were less susceptible to neuronal cell death than their wild-type littermates and displayed less inducible nitric oxide synthase gene expression and reduced O_2_^∙−^ and ONOO^−^ production during ischemia [20, 21].

In the present study, we investigated the therapeutic time window of edaravone on TBI and oxidative metabolite generation in mice following a controlled cortical impact (CCI). We also evaluated the temporal profile of ROS production, oxidative stress, and neuronal cell death in order to estimate the relationship between the effect of edaravone on brain damage and the sequence of events leading to ROS generation following CCI.

## 2. Materials and Methods

### 2.1. Animals and CCI Model

**Figure 1:**
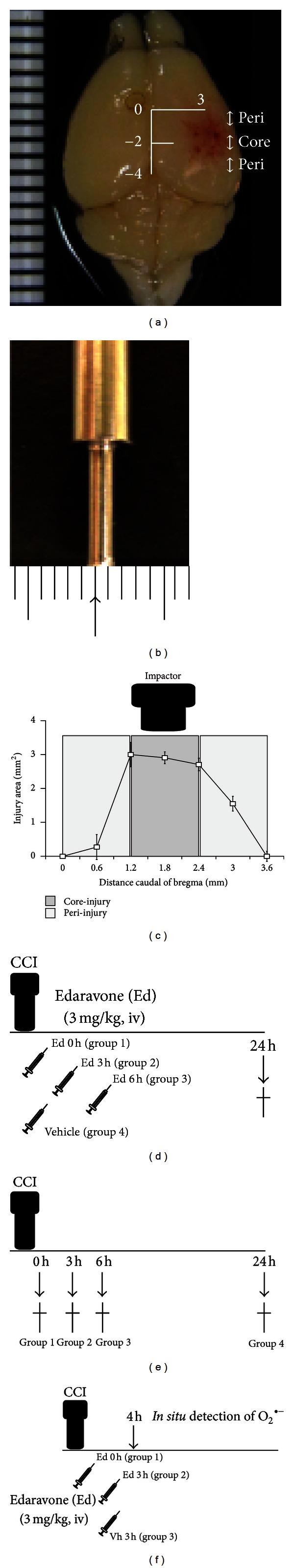
Establishment of TBI model and experimental protocol. (a) Brain images following TBI. The contusion was conducted over the right parietotemporal cortex 2.0 mm caudal of bregma and 3.0 mm lateral of the midline. (b) High power images of the CCI device showing a rounded tip of approximately 1.2 mm in diameter. (c) Coronal cryosections (thickness 50 *μ*m) from bregma to approximately 3.9 mm caudal of bregma encompassing the injury region were used to determine the injury area. The core-injury area was defined as the direct impact region 1.2 mm to 2.4 mm caudal of bregma. The peri-injury area was defined as being <1.2 mm and >2.4 mm caudal of bregma. (d) The free radical scavenger edaravone was injected intravenously into the jugular vein following CCI. To determine the possible therapeutic time window, edaravone (3.0 mg/kg, *n* = 10 for each time point) was administered either immediately (0) or 3 or 6 hours following CCI. As a vehicle-treated control group, saline was administered immediately (0 hour) following CCI (*n* = 9). (e) Temporal profiles of ROS, oxidative stress, and neuronal death in the brain following CCI were determined immediately (0) and at 3, 6 and 24 hours after CCI (*n* = 3). (f) Edaravone treatment to ROS was investigated. Edaravone was administered to group 1: immediately (0 hour) or group 2: 3 hours after the CCI and *in situ detection of *O_2_^∙−^ were performed 4 hours post-CCI. As the control, Group 3: vehicle was administered 3 hours post-CCI and *in situ detection of *O_2_^∙−^ was also performed 4 hours following CCI.

All experimental procedures involving animals were approved by the Institutional Animal Care and Use Committee of Showa University (#00158). Young adult male C57/BL6 mice (8–12 weeks of age, 20–26 g per body weight) were anesthetized with 2% sevoflurane in N_2_O/O_2_ (70%/30%) and positioned in a computer-guided stereotaxic system (Leica Angle Two, Leica Microsystems, Wetzlar, Germany) incorporating an electromagnetic CCI device (Benchmarked Stereotaxic Impactor, Leica Microsystems). Following a midline scalp incision, a 4 mm^2^ opening was made in the skull 3 mm lateral and 2 mm caudal of bregma, thereby exposing the right parietotemporal cortex. A CCI was carried out at a depth of 1.0 mm from the dura mater at a stroke velocity of 3.7 m/second, using an impact device with a rounded tip of approximately 1.2 mm in diameter (Figures 1(a) and 1(b)) [22].

After removing hemorrhaged blood resulting from the impact, the skull was covered with 4 mm diameter artificial dura (GORE Preclude, W. L. Gore & Associates, Newark, NY, USA) and a 5 mm diameter artificial bone plate made from dental cement (GC Fuji I, GC Corporation, Tokyo, Japan). The core body temperature of the mice was maintained at 37°C during the surgery.

### 2.2. Experimental Design

We performed 3 experiments as given in the following.

*Experiment  1*. The animals were divided into 4 experimental groups to determine the possible therapeutic time window of edaravone (3.0 mg/kg, *n* = 10 for each group). Group 1 (Ed 0 h): edaravone was administered immediately (0 hour) after the CCI. Group 2 (Ed 3 h): edaravone was administered 3 hours following CCI. Group 3 (Ed 6 h): edaravone was administered 6 hours following CCI. Group 4, vehicle (Vh): this served as the control group in which normal saline was given immediately (0 hour) following CCI (*n* = 9). All animals were sacrificed 24 hours following CCI (Figure 1(d)). 

*Experiment  2*. Mice (*n* = 3 in each group) were divided into 4 groups. They were sacrificed group 1: immediately (0), group 2: 3 hours, group 3: 6 hours, and group 4: 24 hours after CCI to investigate the temporal profiles of ROS, oxidative stress, and neuronal death in the brain (Figure 1(e)). 

*Experiment  3*. Three groups were set (*n* = 3 in each group) to investigate the effect of edaravone treatment to ROS (Figure 1(f)). Group 1 (Ed 0 h): edaravone was administered immediately (0 hour) after the CCI and *in situ detection* of O_2_^∙−^ was performed 4 hours after CCI. Group 2 (Ed 3 h): edaravone was administered 3 hours following CCI and *in situ detection of *O_2_^∙−^ was performed 4 hours following CCI. Group 3 (Vh 3 h): vehicle was administered 3 hours after CCI and *in situ detection of *O_2_^∙−^ was also performed 4 hours following CCI as the control group.

### 2.3. Administration of Edaravone

Edaravone, a free radical scavenger, was a gift from Mitsubishi Tanabe Pharma (Osaka, Japan). Animals were placed in the supine position and anesthetized with sevoflurane administered by inhalation through a face mask. The skin over the left clavicle was incised to expose the left jugular vein. Edaravone dissolved in saline was slowly administered at a dosage of 3.0 mg/kg body weight (100–150 *μ*L volume) into this vein.

### 2.4. Tissue Preparation

Under sodium pentobarbital (50 mg/kg, i.p.) anesthesia, the animals were transcardially perfused with 0.9% NaCl followed by 2% paraformaldehyde (PFA) in 50 mM phosphate buffer (pH 7.2). The brain and skull were then removed intact and postfixed in 2% PFA overnight, after which the skull was carefully removed, and the brain was immersed in 20% sucrose for 2 days for cytoprotection. The brain was next frozen in liquid nitrogen-chilled 2-methylbutane and coronally cryosectioned at a thickness of 50 *μ*m from bregma to approximately 3.9 mm caudal of bregma, thus ensuring coverage of the injured region (Figure 1(c)). The sections were then immediately immersed in PBS containing 0.1% Tween 20 (PBST) for subsequent histological assessment.

### 2.5. Fluoro-Jade B and Toluidine Blue Staining

Fluoro-Jade B (FJB) staining was used to detect degenerating neurons as previously reported, with only minor modifications [23, 24]. A series of sections was collected at 600 *μ*m intervals from 0 to 3.6 mm caudal of bregma (7 sections per mouse) and mounted on poly-L-lysine-coated glass slides. After air drying, the slides were incubated with freshly prepared 0.06% potassium permanganate for 15 min and rinsed with distilled water. The sections were then immersed in 0.0005% FJB (Millipore, Billerica, MA, USA) in a dark room for 30 min, after which they were completely air-dried before being immersed in xylene and enclosed in malinol (Muto Pure Chemicals, Tokyo Japan). A second series of adjacent sections was stained with toluidine blue (TB) [25]. The sections were observed with a fluorescence microscope (Biozero 8100, Keyence, Osaka, Japan).

### 2.6. Measurement of Lesion Volume

FJB- and TB-stained sections were used to semiquantify the injury area based on sections that displayed FJB labeling or little or no TB staining. Some sections, particularly those near the injury core, lacked part of the neocortex due to severe tissue damage; this area was also included in our calculations of lesion volume. The outlines of the affected regions were traced, and the areas were calculated using NIH Image software. The injury volume was then determined by summing these areas. This was performed by an investigator who was blinded to the experimental groups.

We further defined core- and peri-injury areas. Given that the diameter of the impact tip was around 1.2 mm, we defined the core-injury area as encompassing the region 1.2 mm to 2.4 mm caudal of bregma, with the peri-injury area surrounding this (Figure 1(c)).

### 2.7. Immunostaining of Nitrotyrosine (NT)

Another series of sections at 600 *μ*m intervals (6 sections per animal) was used to label for nitrotyrosine (NT), a peroxynitrite (ONOO^−^) oxidative metabolite, by free-floating immunohistochemistry. After immersion in 0.3% H_2_O_2_, the sections were incubated in 5% normal horse serum and immersed overnight with a polyclonal affinity-purified rabbit anti-NT antibody (1 : 1000, Upstate Biotechnology, Lake Placid, NY, USA). The sections were then incubated with biotinylated goat anti-rabbit IgG (1 : 200, Santa Cruz Biotechnology, Santa Cruz, CA, USA), followed by an avidin-biotin complex solution (Vector, Burlingame, CA, USA) using diaminobenzidine (Vector) as a chromogen. The area of dark brown NT-immunopositive staining as well as that of the severely damaged core-injury region as determined using NIH Image software and the injury volume calculated by summing these areas.

To determine the identity of the NT-positive cells, we colabeled for various cell markers. After blocking, the sections were coincubated with anti-NT antibody and either monoclonal mouse anti-NeuN antibody (1 : 2000, a neuronal marker, Millipore) or monoclonal mouse anti-glial fibrillary acidic protein (GFAP) antibody (1 : 2000, an astroglial marker, Sigma, St. Louis, MO, USA), followed by the secondary antibodies Alexa 488-conjugated goat anti-rabbit IgG antibody (1 : 400) and Alexa 546-conjugated goat anti-mouse IgG antibody (1 : 800, Molecular Probes, Eugene, OR). Nuclei were stained with 4,6-diamidino-2-phenylindole dihydrochloride (DAPI, 1 : 10,000; Roche, Mannheim, Germany).

### 2.8. *In Situ* Detection of O_2_^∙−^

Hydroethidium (HEt) rapidly penetrates into the brain parenchyma, reacts with O_2_^∙−^, and generates ethidium (Et) which can be detected at an emission wavelength of 510–550 nm [26, 27]. Mice were anesthetized with 2% sevoflurane in N_2_O/O_2_ (70%/30%) and were administered 1.0 mg/mL HEt solution (in 0.9% NaCl containing 1% DMSO) into the left jugular vein. Fifteen minutes after HEt infusion, the animals were perfuse-fixed and their brains frozen and sectioned. To identify the Et-positive cells, some sections were also stained with primary antibody for NeuN or GFAP. All sections were nuclear-stained with DAPI. Fluorescence was detected using an Axio Imager optical sectioning microscope with an ApoTome slider module.

### 2.9. Statistical Analysis

Data were expressed as mean ± SEM. Statistical comparisons were made by Student's* t*-test for two groups and by one-way ANOVA followed by *Tukey-Kramer* tests for multiple group comparisons. A value of  *P* < 0.05 was considered to indicate statistical significance.

## 3. Results

### 3.1. Edaravone Has a Therapeutic Time Window of 6 Hours for the Treatment of TBI

**Figure 2:**
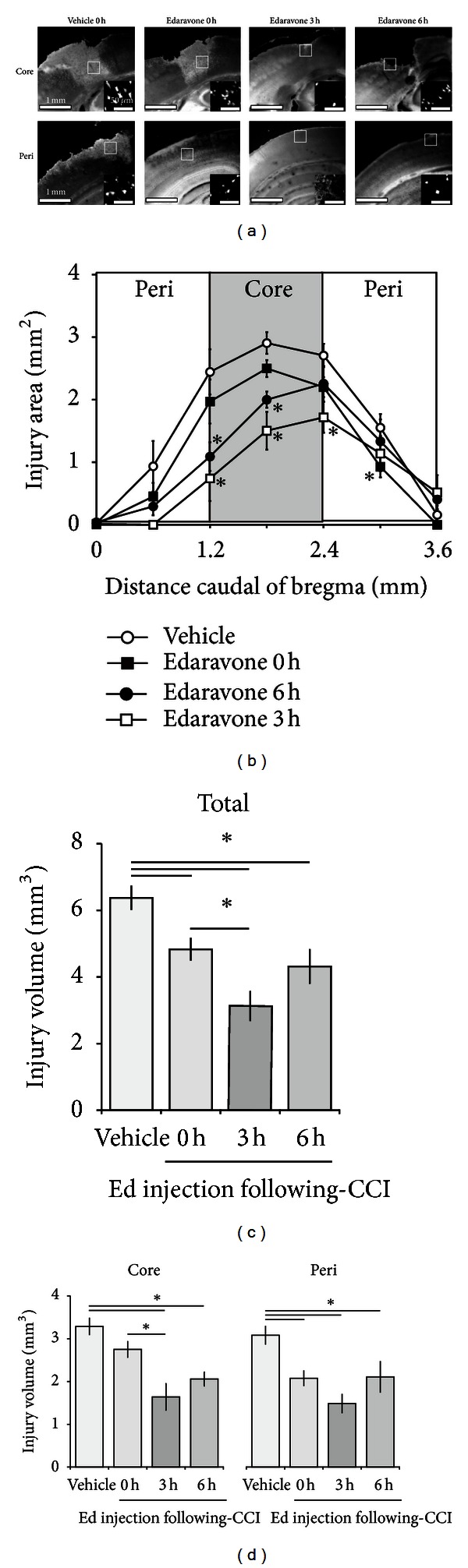
Effect of edaravone on TBI following CCI. (a) Representative core- (*upper*) and peri-injury (*lower*) images of FJB staining at 24 hours following CCI. Edaravone was injected 0, 3, or 6 hours following CCI; vehicle treatment occurred at 0 hours. The inset shows a higher power view of the boxed region in the main image. (b) The TBI area was semiquantified for each experimental group. Edaravone (*n* = 10) treatment at 3 and 6 hours led to a significant decrease in the core-injury area compared with the vehicle-treated control (*n* = 9). The injury volume was calculated by integration of the TBI areas and expressed as total (c) or core- and peri-injury (d) volumes. Data are expressed as mean ± SEM. Asterisk (∗) indicates a significant difference between groups based on *Tukey-Kramer* tests (*P* values are described in the text).

**Figure 3:**
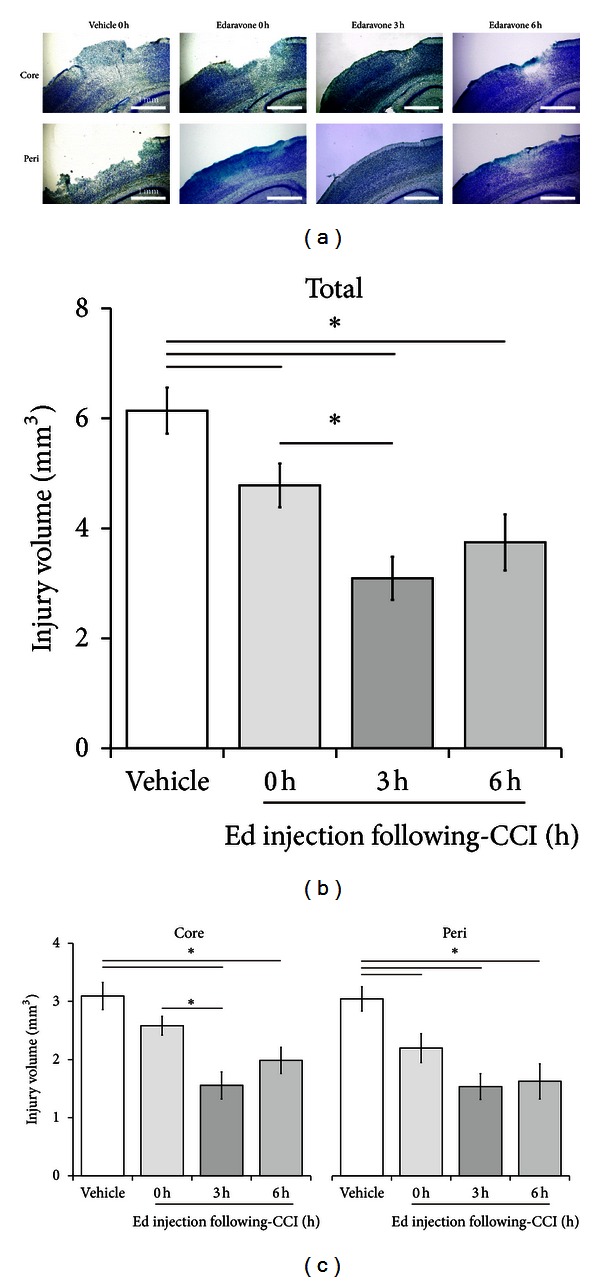
Effect of edaravone on TBI after CCI based on TB staining. (a) Representative core- (*upper*) and peri-injury (*lower*) images of TB staining 24 hours after CCI. The region with little or no staining was defined as the injury area. Edaravone (*n* = 10) was injected at 0, 3, or 6 hours and vehicle (*n* = 9) at 0 hours after CCI. The total (b) or core- and peri-injury (c) areas were semiquantified in each experimental group. Data are expressed as mean ± SEM. Asterisk (∗) indicates a significant difference between the groups based on *Tukey-Kramer* tests.

We commenced by investigating the effect of edaravone and the optimal therapeutic time window for its administration in our experimental TBI model. Animals were administered either vehicle (0 hours; *n* = 9) or 3.0 mg/kg edaravone (0, 3, or 6 hours after CCI) (*n* = 10) (Figure 1(d)) and the efficacy of this treatment was evaluated at 24 hours based on FJB (Figure 2) and TB (Figure 3) staining. 

One vehicle-treated mouse died during the experiment. The total injury volume calculated from FJB staining was significantly reduced in edaravone-treated animals (0 h: 4.83 ± 0.32 mm^3^, *P* < 0.01; 3 h: 3.13 ± 0.43 mm^3^, *P* < 0.001; 6 h: 4.31 ± 0.50 mm^3^, *P* < 0.001) compared with that in the vehicle-treated cohort (0 h: 6.38 ± 0.34 mm^3^). Interestingly, the total injury volume in mice treated with edaravone at 3 hours was significantly less than that which occurred in those treated at 0 hours (*P* < 0.05), with the former animals also displaying the strongest neuroprotection observed across all groups. No significant differences in injury volumes were recorded between the 0- and 6-hour or the 3- and 6-hour treatment groups (Figure 2). 

To confirm these results, we determined the injury volume based on TB staining and obtained similar results (Figure 3). The total injury volume detected by TB staining was significantly reduced in the edaravone-treated groups (0 h: 4.78 ± 0.40 mm^3^, *P* < 0.05; 3 h: 3.09 ± 0.39 mm^3^, *P* < 0.001; 6 h: 3.75 ± 0.51 mm^3^, *P* < 0.01) compared with the vehicle-treated controls (0 h: 6.14 ± 0.42 mm^3^), with the best result again being obtained for the 3-hour treatment group compared with the 0-hour group (*P* < 0.05).

To estimate which region of the affected tissue edaravone rescued from cell death, we also analyzed the volume of the core- and peri-injury areas separately using FJB staining (Figure 2(d)). The animals treated with edaravone at 0 hours had a significantly lower peri-injury volume compared with controls treated with vehicle at the same time point (2.08 ± 0.17 mm^3^ versus 3.09 ± 0.21 mm^3^, *P* < 0.01), but no significant difference was observed for the core-injury volume (2.75 ± 0.18 mm^3^ versus 3.29 ± 0.19 mm^3^). In contrast, edaravone treatment at both 3 and 6 hours significantly reduced the TBI-induced volumes in both the core- (3 h: 1.64 ± 0.31 mm^3^, *P < *0.001; 6 h: 2.06 ± 0.16 mm^3^, *P* < 0.05) and peri- (3 h: 1.49 ± 0.21 mm^3^, *P < *0.001; 6 h: 2.11 ± 0.36 mm^3^, *P* < 0.001) injury sites. To be more specific concerning the core injury area, edaravone treatment at both 3 and 6 hours rescued the frontal region to a greater extent than that seen for the 0-hour treatment (Figure 2(b)). Notably, the core-injury volume following edaravone administration at 3 hours was significantly less than that measured in response to edaravone treatment at 0 hours. The results for TB staining were similar to those for FJB staining (Figure 3). 

### 3.2. Edaravone Treatment Suppresses Oxidative Stress following TBI

**Figure 4:**
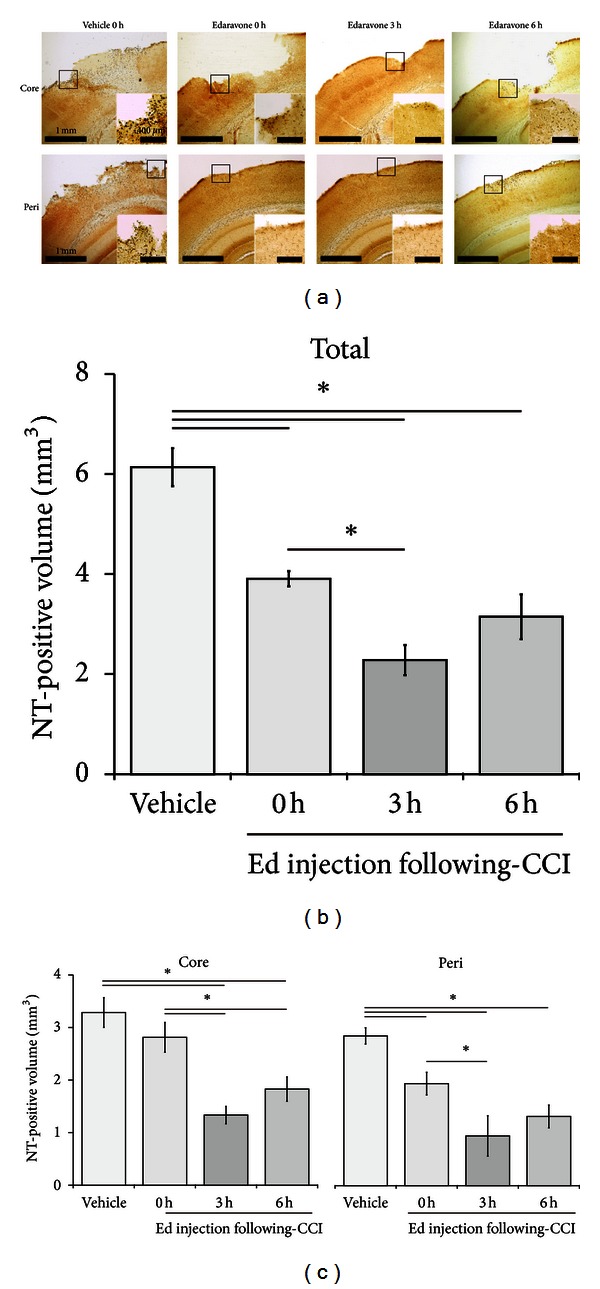
Effect of edaravone on oxidative stress following CCI. (a) Representative core- (*upper*) and peri-injury (*lower*) images of NT staining at 24 hours following CCI. Edaravone (*n* = 10) was injected at 0, 3, or 6 hours following CCI; vehicle (*n* = 9) treatment occurred at 0 hours. The inset shows a higher power view of the boxed region in the main image. The injury volume was calculated by integration of the TBI areas (based on seven 50 *μ*m coronal sections at 500 *μ*m intervals) and expressed as total (b) or core- and peri-injury (c) volumes. Data are expressed as mean ± SEM. Asterisk (∗) indicates a significant difference between groups based on *Tukey-Kramer* tests (*P* values are described in the text).

The previous results revealed that edaravone reduces the area affected by TBI and that it can exert a therapeutic effect when administered up to 6 hours following injury. As previous studies have reported that edaravone acts as a radical scavenger and reduces oxidative stress in a number of diseases [28–30], we therefore next investigated whether the effect of edaravone in our model was due to the suppression of oxidative stress. To assess this, we determined the NT-positive volume 24 hours after CCI in animals treated with either vehicle (*n* = 9) at 0 hours or edaravone (*n* = 10) at 0, 3, or 6 hours (Figure 1(d)). The total NT-positive volumes for the edaravone-treated groups at 0 hours (3.91 ± 0.15 mm^3^, *P < *0.001), 3 hours (2.28 ± 0.30 mm^3^, *P < *0.001), and 6 hours (3.15 ± 0.45 mm^3^, *P < *0.001) were significantly less than those of the vehicle-treated control mice (6.14 ± 0.38 mm^3^) (Figure 4). There was also a marked difference between the 0- and 3-hour edaravone treatment groups (3.91 ± 0.15 mm^3^ versus 2.28 ± 0.30 mm^3^, *P < *0.001). Similar results were obtained for the core- and peri-injury volumes of the animals treated with edaravone at 3 hours (core: 1.33 ± 0.16 mm^3^, *P < *0.001; peri: 0.95 ± 0.38 mm^3^, *P < *0.001) and 6 hours (core: 1.83 ± 0.23 mm^3^, *P < *0.01; peri: 1.32 ± 0.22 mm^3^, *P < *0.01) compared with the vehicle-treated controls (core: 3.29 ± 0.28 mm^3^; peri: 2.85 ± 0.15 mm^3^). However, 0-hour edaravone treatment only produced a significant (*P < *0.05) decrease in the NT-positive peri-injury volume (core: 2.81 ± 0.28 mm^3^; peri: 1.94 ± 0.22 mm^3^). Moreover, the animals treated with edaravone at 3 hours showed significantly greater neuroprotection at both the core- and peri-injury sites versus the 0-hour treatment group (Figure 4). 

### 3.3. Neurodegeneration in the Core Injury Area Occurs at 6 Hours following CCI and Spreads to Peri-Injury Area with Time

**Figure 5:**
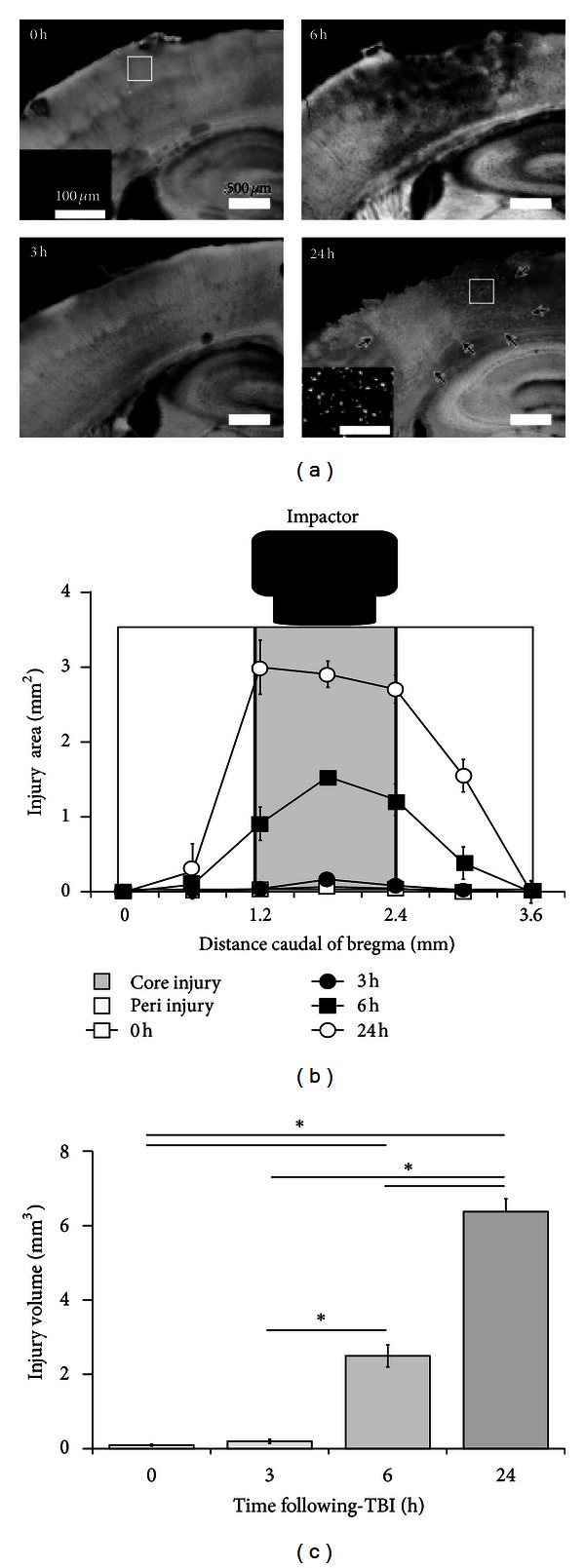
Detection of neuronal cell death by FJB labeling after CCI. (a) Neuronal cell death, as indicated by FJB labeling, increases in a time-dependent manner after CCI (*n* = 3 at each time point). No FJB-positive cells were observed at 0 (also see inset) and 3 hours after CCI; however, by 6 and 24 hours (also see *inset*), FJB labeling and an increase in the area of cortical disruption produced by the contusion were observed. (b) Seven 50 *μ*m coronal sections at 500 *μ*m intervals were used to semiquantify the area of FJB immunoreactivity, together with the area of cortical disruption produced by the TBI. This analysis revealed a marked increase in the affected area at 6 and 24 hours. Data are expressed as mean ± SEM (*n* = 3). (c) A significant increase in the TBI volume was observed 6 and 24 hours following CCI. Data are expressed as mean ± SEM (*n* = 3). Asterisk (∗) indicates a significant difference between groups based on *Tukey-Kramer* tests (*P* values are described in the text).

We next determined the time course (Figure 1(e)) of neurodegeneration following CCI (*n* = 3 for each time point). Only a few FJB-positive cells were present in the core-injury area 3 hours after CCI. By 6 hours, however, diffusely scattered FJB-positive cells were observed not only in the core injury area but also in the peri-injury area. By 24 hours, both the number of FJB-positive cells and the size of the affected area had increased even further (Figure 5). The injury volume was significantly greater at 6 (2.49 ± 0.30 mm^3^, *P < *0.01) and 24 hours (6.38 ± 0.34 mm^3^, *P < *0.01) compared with 0 (*n* = 3, 0.10 ± 0.03 mm^3^) and 3 (0.19 ± 0.05 mm^3^) hours (Figure 5(c)).

### 3.4. Oxidative Metabolites Increase 6 Hours after CCI in Neurons

**Figure 6:**
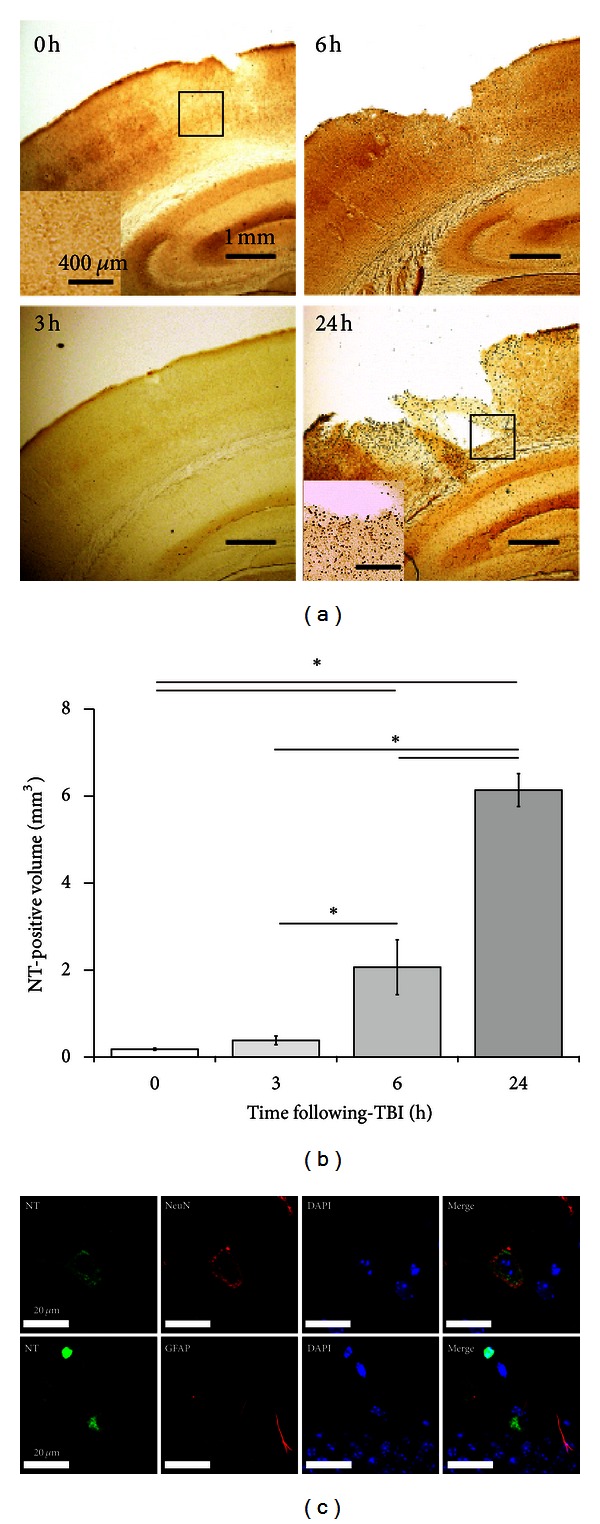
Detection of oxidative stress by NT labeling following CCI. (a) Very few NT-immunopositive cells were observed at 0 (also see *inset*) and 3 hours following CCI; however by 6 and 24 hours (also see inset) this number, and the area of cortical disruption produced by the contusion, had increased markedly. (b) Semiquantification of the NT-positive volume revealed a significant increase at 6 and 24 hours after CCI. Data are expressed as mean ± SD (*n* = 3). Asterisk (∗) indicates a significant difference between groups based on *Tukey-Kramer* tests (*P* values are described in the text). (c) Multiple immunofluorescence staining of NT and cell markers. The NT-positive staining overlapped with that of the neuronal marker, NeuN (*green, upper panel*), but not with the astroglial marker, GFAP (*green, lower panel*). The sections were also counterstained with the nuclear dye DAPI (*blue*).

We subsequently investigated the temporal profile (Figure 1(e)) of oxidative stress by using NT immunostaining after CCI (*n* = 3 for each time point). Minimal immunoreactivity was observed immediately after CCI (0 h). However, dark brown NT staining began to appear from 6 hours following CCI in the neocortex around the epicenter of the impact site. By 24 hours, the NT-positive area had expanded and some of the tissue at the impact site had been lost (Figure 6(a)). The volume of the NT-positive region increased significantly in a time-dependent manner after CCI, accounting for 0.18 ± 0.03 mm^3^, 0.38 ± 0.10 mm^3^, and 2.07 ± 0.63 mm^3^ (*P* < 0.05 versus 0 hour) and 6.14 ± 0.38 mm^3^ (*P* < 0.01 versus 0 hour) at 0, 3, 6, and 24 hours after CCI, respectively (Figure 6(b)).

Colabeling with cell markers at the core-injury site indicated that the oxidative stress was occurring mainly in neurons (Figure 6(c)).

### 3.5. ROS Increases in Neurons 3 Hours after CCI

**Figure 7:**
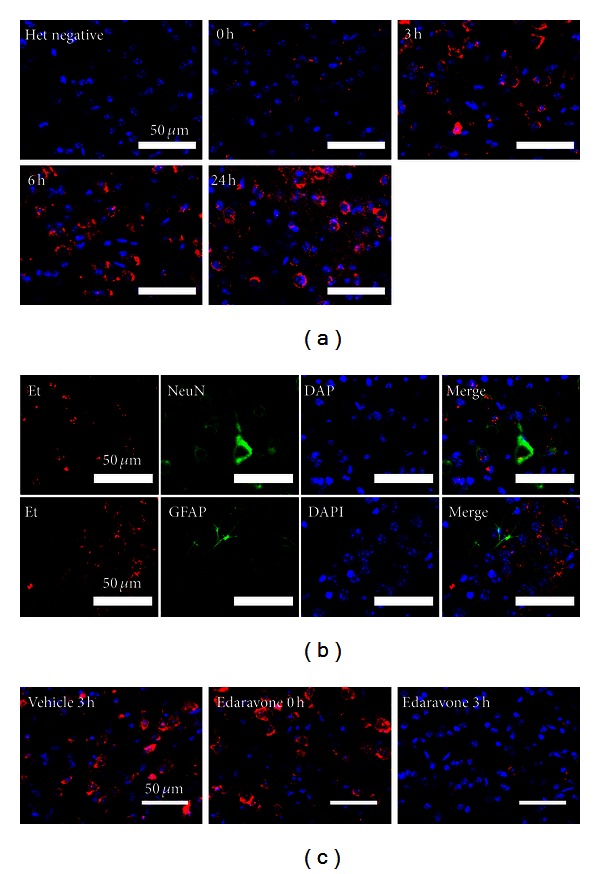
*In situ* detection of ROS as O_2_^∙−^ using Het. (a) No O_2_^∙−^ (Et) signals (red) were detected in control animals. In mice subjected to TBI, a low level of O_2_^∙−^ was observed immediately after CCI. By 3 hours this level had increased markedly, remaining high 6 and 24 hours after CCI. (b) Multiple immunofluorescence staining of O_2_^∙−^ and cell markers. O_2_^∙−^ (Et) signals (*red*) strongly colocalized with the neuronal marker NeuN (*green, upper panel*) and to lesser extent with the astroglial marker GFAP (*green, lower panel*), with nuclei labelled by DAPI (*blue*). (c) The effect of edaravone treatment on O_2_^∙−^ production after CCI (*n* = 3 in each group) is shown. As a control, vehicle was administered 3 hours post-CCI (vehicle 3 h). Edaravone was administered at 0 hours (edaravone 0 h) or 3 hours (edaravone 3 h) after CCI. The production of O_2_^∙−^ was evaluated based on the *in situ* detection of the Et signal (*red*) in the core-injury area 4 hours after CCI. Nuclei (*blue*) were labeled with DAPI.

We next determined the time course (Figure 1(e)) of ROS generation based on the *in situ* detection of O_2_^∙−^ using HEt at 0, 3, 6, and 24 hours following CCI (*n* = 3 for each time point). A low Et signal was initially observed in the core-impact region. However, by 3 hours following CCI, this signal had increased markedly and continued to rise slightly up until the 24-hour time point (Figure 7(a)). Colabeling for Et and various cell markers revealed that the affected cells were mainly NeuN-positive neurons (Figure 7(b)).

### 3.6. Edaravone Suppresses the ROS Production Cycle following CCI

Finally, we also investigated the effect of edaravone treatment 3 hours following CCI on O_2_^∙−^ generation at the core-injury site using HEt injection. Mice (*n* = 3 in each group) were administered edaravone 0 or 3 hours following CCI or vehicle 3 hours following CCI, with the Et signal evaluated 4 hours after injury as a measure of O_2_^∙−^ generation. As illustrated in Figure 7(c), vehicle-treated brains showed a large number of affected cells and a strong Et signal intensity. Fewer cells were affected following edaravone treatment at 0 hours, but the intensity of the Et signal became stronger thereafter. However, the 3-hour treatment resulted in only a few affected cells and a very weak Et signal, suggesting that edaravone administered at this time point suppressed the ROS and oxidative stress cycle and provided a greater neuroprotective effect.

## 4. Discussion

Edaravone is a free radical scavenger approved in Japan for the treatment of stroke. It could be a suitable candidate for treating TBI as well, given the results of several rodent studies showing that this drug is able to decrease neuronal cell death. However, the therapeutic time window of edaravone on TBI has not been examined in detail. In the present study, we have demonstrated that the intravenous injection of edaravone (3 mg/kg) decreased TBI and reduced oxidative stress when administered after a delay of up to 6 hours following CCI. We also determined the temporal profiles of ROS production, oxidative stress, and neuronal cell death in order to understand the relationship between brain damage and the sequence of events underlying ROS generation following CCI.

We previously reported that the intravenous administration of edaravone (3 mg/kg) immediately after TBI suppressed cortical damage [13]. Another study also demonstrated that edaravone injected 2 and 12 hours after TBI decreased neuronal cell loss in the CA3 layer of the hippocampus in a dose-dependent fashion (0.75, 1.5, or 3 mg/kg) [8]. In the present study, we examined the therapeutic time window of intravenously injected edaravone (3 mg/kg) on TBI and showed that edaravone administered for up to 6 hours at least after CCI suppressed the lesion size. Comparisons of lesion sizes following edaravone treatment at 0, 3, and 6 hours after CCI demonstrated that edaravone treatment at 3 hours provided the greatest neuroprotective effect compared with the other treatment groups and that a significant difference was observed compared with treatment at the 0-hour time point. To estimate the extent of the neuroprotective effect, we further compared the lesion size by measuring both core- and peri-injury areas. Edaravone treatment 6 hours after CCI decreased the lesion size significantly both in the peri- and core-injury sites compared with control, but the area was slightly larger than that seen in mice treated with edaravone 3 hours after CCI. Although mice treated with edaravone at the 0-hour time point showed a significantly decreased lesion size in the peri-injury site compared with vehicle-treated animals, no statistically significant differences with respect to the core-injury site size were seen. Furthermore, the size of the core-injury site in the 0 hour edaravone treatment group was significantly greater than that measured in mice treated 3 hours after CCI. In particular, the lesion size in the frontal area was significantly different between these two groups. Observations made with respect to NT immunostaining as an indicator of oxidative stress also demonstrated a similar tendency to that seen with lesion size. 

We subsequently examined the temporal profiles of ROS production, oxidative stress, and neuronal cell death in order to understand the relationship between brain damage and the sequence of events giving rise to ROS generation following CCI. Superoxide detected by HEt was used for the determination of ROS levels because O_2_^∙−^ is initially increased after injury [26], while the oxidative metabolites ONOO^−^ and ^*∙*^OH also contribute to increasing oxidative stress and damage in tissue [11]. The O_2_^∙−^ signal was initially observed 3 hours following CCI and increased with time up to 24 hours. The oxidative stress detected by NT, which is a metabolite of L-tyrosine oxidation by ONOO^−^ [31], was observed at 6 hours following CCI in the core-injury region and increased for up to 24 hours with extension to the peri-injury area. This was reflected in the rise in O_2_^∙−^ production and the concomitant increase in neuronal cell death detected by FJB staining, suggesting that excessive O_2_^∙−^ production after CCI might result in the induction of oxidative stress and neurodegeneration in the brain.

From the results of the temporal profiles of ROS production, oxidative stress, and neuronal cell death, the effects of treatment with edaravone were consistent with the time points which fall before, during, and after the production of ROS, respectively. While a precise explanation for this cannot be given, we suggest that it could have something to do with the half-life of edaravone, which is reported to be approximately one hour [29]. Therefore, the animals treated with edaravone immediately after (0 hour) CCI might actually have had a decreased effective concentration of this drug at the time of maximum ROS production. By 6 hours following CCI, a weaker neuroprotective effect was observed due to the fact that significant oxidative damage and neuronal cell death had already occurred. Nonetheless, treatment at this time point still had a therapeutic effect given that the level of ROS continued to increase for 24 hours. These results suggest that there might be an optimal time for treatment with edaravone to suppress the degree of injury. An experimental study in which mice were exposed to hypobaric conditions for 3 hours after TBI supports our present results; these animals showed exacerbated secondary traumatic injury severity because of a greatly heightened inflammatory response due to the hypobaric condition [32]. We recently found that edaravone improved cerebral blood flow (CBF) after TBI [33]. In this way, CBF in control animals in that study was significantly reduced, probably as a consequence of vasospasm, in the ipsilateral hemisphere of the brain 3 to 6 hours after CCI [33]. Treatment with edaravone significantly ameliorated the CBF. Other studies have reported that edaravone improves blood circulation in the heart [34] and lung [27], suggesting that edaravone treatment may suppress traumatic ischemic injury, as observed in the present study, by ameliorating circulation. 

This finding is important given that most TBIs are associated with falls and motor vehicle accidents. Therefore, in most cases the onset of TBI is clear and patients are transferred to an emergency department within a few hours. From a clinical perspective, it is noteworthy that delayed treatment with edaravone in our study was more efficacious than administration immediately after the CCI; the time delay between the onset of ROS production and neuronal cell death means probably that these cases would fall within a therapeutic time window for the treatment of TBI. At the present time, edaravone (60 mg/day injected intravenously in 2 divided doses) is used in Japan for the treatment of stroke. Therefore, the dosage of edaravone in our study was approximately triple compared with clinical use. Further study is required to determine the minimum dosage of edaravone necessary to suppress ROS production and lesion size in this animal model and for this finding to be translated to the clinical setting whereby edaravone is used to suppress the lesion size in clinical cases of TBI.

Although many studies have reported that oxidative stress contributes to TBI, very few have linked this directly to ROS production in the brain. We have previously reported that both patients suffering a neurological emergency and animals subjected to TBI display increased blood alkoxy-radical levels as detected by an electron spin resonance (ESR) spin trapping method [13, 35]. Based on the rapid elevation of intracellular Ca^2+^ and the impairment of CBF, the source of the O_2_^∙−^ is considered to be primarily the mitochondria [36]. The intracellular O_2_^∙−^ impairs mitochondrial function and induces neuronal cell death. Extracellular O_2_^∙−^ might be produced by NADPH oxidase in microglia/macrophages following CCI [19]. Based on our previous demonstration that mice deficient in one of the subunits of NADPH oxidase, Gp91^phox^, display decreased O_2_^∙−^ levels and that inhibition of Gp91^phox^ by apocynin reduces the severity of TBI *in vivo* [19, 36, 37], it appears that this extracellular O_2_^∙−^ also contributes to the induction of neuronal cell death in response to injury. Therefore, both intra- and extracellularly produced O_2_^∙−^, together with their associated metabolites, may play important roles in the generation of oxidative stress following CCI. 

Many clinical trials of antioxidant agents or radical scavengers have been performed in cases of cerebral infarction, subarachnoid hemorrhage, and TBI [15, 38, 39]. However, the only agent which has been granted approval to date is edaravone [9, 40], even though patients are treated without concomitant monitoring of ROS or oxidative metabolite levels. Human TBI presents as a more complex and diverse condition than animal experimental models. Therefore, care needs to be exercised when determining the likely therapeutic time window of edaravone. We suggest that edaravone and possibly other radical scavengers or antioxidants should be administered in response to ROS generation, meaning that the bedside monitoring of ROS could shed more light on the potential benefit of these agents. Recently, some groups have reported that Overhauser enhanced MRI, which is a double resonance technique, creates images of free radical distribution in small animals by enhancing the water proton signal intensity by means of the Overhauser effect [41]. Although not available for bedside monitoring, this technique could be adapted to directly monitor ROS levels in the brain. We have also previously reported that one particular ROS, the alkoxy radical, is increased both in patients suffering a neuroemergency and in animals subjected to TBI and can be monitored by ESR [13, 35]. These new techniques could represent powerful tools for the diagnosis of ROS production prior to oxidative stress, thereby facilitating more effective treatment. 

## 5. Conclusion

In the present study, we have demonstrated that edaravone suppresses neuronal damage in mice subjected to a CCI, with the greatest effect observed when the drug is given 3 hours after TBI. This time window is consistent with the increase in ROS produced in the cortex after CCI. We therefore suggest that edaravone could prove clinically useful to ameliorate the devastating effects of TBI. To ensure optimal efficacy, however, it is critical that ROS levels are measured concomitantly.
